# Latest progress in tyrosine kinase inhibitors

**DOI:** 10.18632/oncotarget.1836

**Published:** 2014-03-20

**Authors:** Tatiana V. Pospelova, Valery A. Pospelov

**Affiliations:** ^1^ Institute of Cytology, Russian Academy of Sciences, St. Petersburg, Russia; ^2^ Saint Petersburg State University, St. Petersburg, Russia

**Keywords:** cancer, mTOR, kinases, c-Met, Bcr-Abl, senescence

## Abstract

Here we discuss the latest progress in development of some kinase inhibitors such as inhibitors of c-MET, LIM and Bcr-Abl kinases. Importantly, many oncogenic kinases signal via the mTOR pathway, suggesting a common target for drug combinations.

Met tyrosine kinase receptor signaling is activated by its ligand, hepatocyte growth factor/scatter factor (HGF/SF) [1, 2]. MET signaling in turn increases glycolysis, oxidative phosporylation, and tumor blood volume [3]. It was shown that HGF/SF-activated Met increased Ras activity, Erk phosphorylation and cell motility [3]. Tang et al. compared the demographic and clinical characteristics of ovarian cancer patients with MET altered patients and their response to c-Met inhibitors [4]. MET nonsynonomous nucleotide variations and amplification occurred in 7.4% and 3.5% of patients, respectively. MET variations were observed only in white women with high-grade ovarian tumors, whereas amplifications were detected in both black and white women with high-grade serous ovarian primary tumors. MET alterations have been associated with resistance to therapy [5]. Thus, patients exhibiting a MET alteration did not achieve an objective response by a c-Met inhibitor therapy [4]. Preclinical experiments have shown that the simultaneous use of two inhibitors anti-Met and anti-EGFR significantly enhance the effectiveness of tumor growth inhibition [6]. C-MET amplified subpopulation of cells existed prior to anti-EGFR treatment supporting idea co-treatment of patients with Met and EGFR therapies [7]. MET amplification in colorectal carcinomas associated with resistance to cetuximab and panitumumab [8]. Taken together these results provide a strong rationale for the use of Met inhibitors to overcome drug-resistance to EGFR therapies.

Importantly, activators of c-MET are secreted by adipose-derived mesenchymal stem cells, which exacerbate oncogenic behaviour of c-Met-expressing breast cancer cells, creating an inflammatory microenvironment, thus increasing tumor growth and angiogenesis. c-Met expression is a predictive factor of cancer recurrence after autologous fat graft in post-surgery breast cancer patients [9].

Recently nuclear factor κ-B kinase (IKK) was identified a novel signaling mechanism for the regulation of mTORC2. A new inhibitor of IKK Bay 11-7082 interacts with Rictor and regulates the activity complex mTORC2. Rictor phosphorylation at T1135 was also inhibited by the IKK inhibitor Bay 11-7082 [10]. IKK regulates mTORC2 activity including phosphorylating AKT at the serine 473 and actin cytoskeleton reorganization, which is controlled by LIM kinases. The LIM kinases are promising oncotarget in several types of cancer [11-13]. The main substrate of LIM kinase is cofilin, an actin-depolymerizing factor. LIMK1, a modulator of actin and microtubule dynamics, is involved in the mitotic process through inactivating phosphorylation of cofilin [14]. LIMK2 increases resistance to chemotherapeutic agents in neuroblastoma cells by regulating drug-induced cell cycle arrest [15]. A LIMK inhibitor, T56-LIMKi, inhibits LIMK2 with high specificity, while not inhibiting LIMK1 [13]. T56-LIMKi decreases phosphorylated cofilin (p-cofilin) levels and inhibits growth of glioma, schwannoma and pancreatic cancer. T56-LIMKi reduced tumor size and p-cofilin levels in the pancreatic tumors [13]. Also, thioredoxin inhibition is emerging as attractive strategy [16-18] especially employing already existing drugs such as Disulfiram for novel use [16, 19, 20].

Although tyrosine kinase inhibitors have changed therapy of chronic myeloid leukemia (CML), acquired resistance to imatinib, dasatinib or nilotinib, due to BCR-ABL1 kinase mutations, limits the therapy. Also, hematopoietic niche could protect leukemic cells from therapy [21]. Aggoune et al demonstrated that T315I mutants need either compound mutations such as E255K/T315I or a stromal niche to escape from the toxicity of ponatinib [21]. Thus the hematopoietic niche plays a crucial role in conferring resistance to ponatinib, by increasing cell survival and genetic instability [21]. This is especially striking given that chronic myelogenous cells that are resistant to all kinase inhibitors are still highly sensitive to ponatinib [22, 23].

Inhibitors of the mTOR pathway sensitize chronic myeloid leukemia stem cells to nilotinib and restore the response of progenitors to nilotinib in the presence of stem cell factor [24-26]. Inhibitors of mTOR also sensitized tumor cells to other kinase inhibitors [27]. Noteworthy, the mTOR inhibitor rapamycin (sirolimus) also prevents cancer in animal models [28-36]. Rapamycin slows down organism aging [37-58], by preventing cellular geroconversion to senescence [59-70]. And everything that slows down aging in turn prevents cancer because cancer is an age-related disease [71, 72].
